# An analysis of investors’ behavior in Bitcoin market

**DOI:** 10.1371/journal.pone.0264522

**Published:** 2022-03-10

**Authors:** Delia-Elena Diaconaşu, Seyed Mehdian, Ovidiu Stoica

**Affiliations:** 1 Department of Social Sciences and Humanities, The Institute of Interdisciplinary Research, Alexandru Ioan Cuza University of Iasi, Iasi, Romania; 2 School of Management, University of Michigan-Flint, Flint, Michigan, United States of America; 3 Department of Finance, Money and Public Administration, Faculty of Economics and Business Administration, Alexandru Ioan Cuza University of Iasi, Iasi, Romania; University of Almeria, SPAIN

## Abstract

As an emerging digital asset, Bitcoin has been traded for more than a decade, reaching an impressively high market capitalization and continuing to expand its volume of trading at a rapid pace. Many countries have legalized or are considering legalizing a trading platform for this asset, and a set of companies worldwide accept it as a medium of exchange. As a result of this expansion, many studies in finance literature have focused on studying the efficiency of this cryptocurrency. In line with this literature, this paper investigates, using the abnormal returns and abnormal trading volumes methodologies, the dynamics of investors’ reaction to the arrival of unexpected favorable and unfavorable information regarding the Bitcoin market in the context of the three famous hypotheses: the overreaction, the uncertain information, and the efficient market hypotheses. Overall, we find evidence confirming that the Bitcoin market tends to mature over time. More precisely, over the entire analyzed period, investors behave in accordance with the predictions of the uncertain information hypothesis when positive and negative events occur. However, splitting the timespan into sub-periods provides interesting insights. Remarkably in this respect is the fact that starting with the second sub-period, the response of investors in the Bitcoin market supports, in a moderate manner, the postulate of the efficient market hypothesis when favorable events are addressed. Moreover, our findings reveal that during the pandemic period, the efficiency of Bitcoin has increased, thus turning this stressful period into an advantage for this cryptocurrency. This improved market efficiency is also supported by the abnormal trading volume analysis.

## Introduction

The striking development and uniqueness of cryptocurrencies have attracted the attention of market participants in every corner over the last decade. Overall, the galloping development of the market for cryptocurrencies led to a market capitalization of US$2140 billion as of August 2021 [1], thus drawing the attention of policymakers, investors and researchers; and, of course, from this vast list of novel forms of electronic money, Bitcoin is being perceived as having a privileged status. Mostly, the flourishing interest of academia in this relatively new asset class was driven by its unique nature (see [2, 3] for a comprehensive review). To all appearances, among the empirically documented inherent features of these synthetic currencies, it seems that so far several are indisputable, to wit, high volatility [4–6], clustering and long memory of volatility [7–10], the presence of jumps [11–13], high correlation within the crypto market [14–16] but relative isolation from other asset classes in normal times [17–19] and increased contagion in severe turbulent times [20–22], etc. The most disputable characteristics of this atypical asset refer to the investment or currency potential and its ability to act or not as a safe haven. In this regard, [23, 24] argue that it is unlikely to consider the most popular digital currency as a safe haven investment or a medium of exchange, especially in the short-term [25] and especially during the coronavirus pandemic [26, 27]. These findings are at odds with those of [28], which suggests that Bitcoin can be considered akin to ‘virtual gold’ in periods where stock markets have high volatility and especially in the short-term and during the Covid period [29]. Moreover, this cryptocurrency can be regarded as a currency, one that is placed between gold and the dollar [30]. Along these lines, a more balanced evidence comes from [31], who states that Bitcoin represents a unique asset that possesses properties of both a standard financial asset and a speculative one, and from [32], who posit that Bitcoin does not behave like traditional currencies or commodities but should be treated as a technology platform and that this crypto market is much more mature and much less speculative than has been previously suggested.

Treating Bitcoin as a type of virtual financial asset is at the origin of the vast recent literature testing the dominant paradigm in financial economics–the Efficient Market Hypothesis (EMH) of [33]–on Bitcoin prices. Firstly, the semi-strong efficiency of Bitcoin was of interest in [34] who showed, using an event study methodology, that the cryptocurrency has become more efficient over time concerning its events, but it is undoubtedly inefficient in conjunction with the monetary policy events. Contrary to these results, [35] found that the cryptocurrency market is further maturing through interactions with macroeconomic factors.

Secondly, this lack of consensus is also to be found within the results related to the weak-form efficiency of Bitcoin. As a parenthesis, we might mention that there are findings that contradict the (in)efficiency of the crypto market since its long memory feature evolves with time, thus validating the adaptive market hypothesis [36, 37]. On the one hand, the first and widely known study on this subject is done by [38], who concluded that the Bitcoin market was generally inefficient in the period 2010–2016 but maybe in the process of moving towards an efficient market. In contrast, [39] validated the weak efficient hypothesis within the same timespan as the latter study, reasoning that everything is not so negative about Bitcoin (see also [40]). Additional evidence for the trend towards informational efficiency of this digital currency in recent times comes from [7, 41–43]. In this same context, [44] highlighted several periods with significant anti-persistent memory in the BTC-USD series.

On the other hand, compelling evidence against the EMH–especially in recent times–was provided by [45–47]. In other words, the authors pointed out that Bitcoin does not become more efficient over time, but on the contrary, it becomes more inefficient. This unveils that the long memory found in returns can help investors capture speculative profits. Therefore, the analysis of the so-called market anomalies where certain patterns in price behavior makes prices predictable in the short run is quested. More precisely and strongly related to our subject, it seems that the overreaction and the asymmetric behavior of investors have gained special attention in recent literature. In this respect, [48] detected overreactions in the cryptocurrency market for the period 2013–2017. Furthermore, the authors concluded that this price behavior does not give rise to exploitable profit opportunities due to transaction costs; therefore, it cannot be seen as evidence against the EMH. In the same vein, [49] found similar overreaction patterns for twelve cryptocurrencies but concluded that they are suitable for trading price reversals. Besides, the authors argue that hourly returns during the day of positive abnormal returns are significantly higher than those during the average positive day and vice-versa. This result was similar to the findings involving the overreaction behavior of the Bitcoin market of [50]. In addition, the authors found that investors overreacted during days of sharp declines in the Bitcoin price and weeks of market rallies. Also, the same asymmetrical overreaction of the investor on this crypto market was found by [51–53]. Briefly, the authors emphasized that the market reaction to negative events is stronger than to positive events; that is to say, the cryptocurrency market is inefficient.

Summing up, the recent empirical evidence does not provide a common picture regarding the (in)efficiency of the Bitcoin market. Given the discordant, even opposing results related to price behavior on this market that is not too far away from its infancy phase, our work seeks to contribute to the fact-finding concerning the time-varying efficiency of cryptocurrencies and their deviations. Also, given the impressive number of studies in the present day on digital assets, it seems that the analysis of the dynamics of Bitcoin’s behavior is a state-of-the-art of the academic perimeter. In addition, given the ever-changing features of this emerging asset, we believe this market provides a perfect setting to study investor behavior, especially in the present time when this digital asset is in the spotlight.

In a nutshell, the Overreaction Hypothesis (OH) [54, 55] postulates that investors overreact to the arrival of unexpected information. That is, they set the prices above their intrinsic value in reaction to the arrival of unexpected favorable information and below intrinsic value in reaction to the arrival of unexpected unfavorable information. However, the subsequent price movements are corrective downward in the case of favorable information and corrective upward in the case of unfavorable information. The Uncertain Information Hypothesis (UIH) [56] posits that due to the uncertainty generated by the arrival of unexpected information—no matter favorable or unfavorable—investors behave rationally and set the prices below their intrinsic values. However, as the dust of uncertainty settles, the market observes an upward corrective pattern, and prices increasingly approach their intrinsic values. Hence, the importance of studying the efficient market assumption and its anomalies within the Bitcoin market lies in the fact that this novel and emerging asset that, not surprisingly, is defined as excessively volatile, can contribute to the performance of portfolio diversification and can be profitably exploited by using an appropriate trading strategy. We note that the OH and the UIH, following the arrival of unexpected information, predict a process of corrective price patterns.

The present paper contributes to the current and growing literature on cryptocurrency by examining the reactions of Bitcoin investors to the arrival of unexpected favorable and unfavorable information, in general, market surprises. More specifically, we study Bitcoin investors’ reactions in the context of the Overreaction Hypothesis, the Uncertain Information Hypothesis, and the Efficient Market Hypothesis (EMH) over almost its entire existence. To achieve our goal, we have considered several objectives. Firstly, to better capture the dynamics of investors’ behavior on the cryptocurrency market we split our sample into three periods. In this manner, the division into three different periods–its appearance, development, and COVID19 period–made it possible to highlight particular degrees of (in)efficiency for each time segment. Secondly, we employed a simple abnormal return methodology for all periods to assess whether Bitcoin’s efficiency is evolving over time. Then, we performed an analysis of the abnormal trading volume because in the financial market literature this is a well-known practice [57–59]. To date, a very limited number of studies related to cryptocurrencies have investigated their abnormal volume (see e.g., [60–62]). However, none of these studies analyzed both the abnormal return and the trading volume to assess the efficiency of Bitcoin in the light of the three hypotheses mentioned above.

Overall, our paper contributes to the current rare literature related to both the impact of the ongoing COVID19 pandemic on the efficiency of the Bitcoin market and the investigation of the abnormal trading volume of this special asset. Briefly, the results of the analysis of abnormal returns and trading volume suggest that, over time, the Bitcoin market has shown some signs of maturity and a tendency towards the onset of efficiency. Even though cryptocurrencies are considered atypical assets, our results are consistent with the notion that financial markets may be inefficient in the early stages of their existence. Still, they become more efficient as more investors participate in such markets and they grow.

The remainder of the paper is presented as follows: the second section explains the data and the methodology. The empirical results are presented and discussed in the third section, and the last section concludes.

## Data and methodology

To explore the abovementioned (in)efficiency assumptions, we focus our analysis on the largest cryptocurrency by market capitalization, namely, Bitcoin. As Bitcoin markets are highly integrated and, thus, Bitcoin is a universal asset globally determined [62], we decided to conduct our analysis merely based upon the prices and trading volume expressed in USD collected at a daily frequency from http://data.bitcoinity.org (e.g., [23, 63, 64]). The data covers a period from January 1, 2011, to August 12, 2021. Several descriptive statistics of the raw data–Bitcoin prices and volume data–for the analyzed time span is reported in S1 and S2 Appendices.

The methodology adopted in our paper is different from the traditional event study approach. More specifically, we employed a method proposed by [56] and used by [65] and [66] to calculate post-surprises cumulative abnormal returns and trading volumes.

To start, we computed daily returns as the first natural logarithmic difference of the closing value of Bitcoin price. To test for the stationarity of the returns series, we conducted the ADF, KPSS, and Phillips-Perron tests, and the results of these tests indicated that the returns are stationary and integrated of order zero. In line with the literature on trading volume [57, 60] but also considering the fact that the raw trading volume data is highly non-normal and, at least in the first period, there are several days where no trading place appear in the database, we performed the event study using the transformation natural logarithm of (trading volume + 1).

We chose to form four samples, i.e., an interval that covers the entire timespan, from January 1, 2011, to August 12, 2021 (henceforth the whole period), an interval that ranges from the beginning of the period to December 2013 (henceforward first period), an interval that ranges between January 1, 2014 and March 10, 2020 (henceforth second period), and one from March 11, 2020 –the announcement date of the outbreak of COVID19 global pandemic by the WHO–until the end of the sample (henceforth COVID19 period). The reason for splitting the data into three periods is twofold. Firstly, the graph of the return maps a break in the data series in December 2013, and the Quandt-Andrews Breakpoint and the Multiple Breakpoint tests indicate a break within that month. In addition, the empirical evidence suggests that the crashes that occurred after 2014 have been smaller in magnitude and less susceptible. It seems that only starting with 2014 have the market liquidity and efficiency improved, being, thus, perceived as a sign of a maturing market [7, 34]. Nonetheless, this alleged maturity is still not set in stone [47]. Therefore, by dividing the sample, we aimed to emphasize the dynamics of Bitcoin efficiency and, hence, to find out whether, indeed, this popular cryptocurrency is on its way towards maturing. Secondly, the last analyzed period that encompasses the ongoing COVID19 pandemic was chosen to examine the behavior of Bitcoin during an unexpected global catastrophic event. However, the last year and a half comprise not only the pandemic period, but also some important announcements of a growing number of large corporations such as Tesla, PayPal Holdings Inc, French insurer AXA, Bank of NY Mellon Corp, etc. related to Bitcoin acceptance as a form of payment. These (un)foreseen events have raised the trading activity of Bitcoin, which has led to its impressive development.

Table 1 displays the summary statistics of daily returns and log trading volume for Bitcoin during the four periods under study. As can be easily seen and as expected, the highest mean return and market volatility, measured by standard deviation, are exhibited during the first period of analysis. Likewise, and as expected, the deviation from normality is pronounced in all return series of the analyzed samples, having negative asymmetric and leptokurtic distributions, as the values recorded are quite far from those corresponding to a Gaussian distribution. In terms of trading volume, the highest value of the mean is recorded in the last period, while the highest volatility is registered in the entire and first analyzed interval. Also, it is noteworthy the fact that the skewness approaches the zero value over the studied periods, whereas the kurtosis is decreasing, indicating that extreme outliers are less than those of the normal distribution.

**Table 1:** Summary statistics for Bitcoin daily returns and log trading volume.

Period	Log return					Log trading volume				
	Mean	Median	St.dev.	Skewness	Kurtosis	Mean	Median	St.dev.	Skewness	Kurtosis
Whole period (1.01.2011–12.08.2021)	0.307	0.210	4.291	-0.525	20.502	16.892	17.444	3.669	-1.329	4.982
First period (1.01.2011–31.12.2013)	0.714	0.469	6.285	-0.622	14.596	12.356	12.873	3.436	-0.794	3.770
Second period (1.01.2014–10.03.2020)	0.105	0.071	3.097	-0.192	7.571	18.269	18.094	1.536	0.242	1.923
COVID19 period (11.03.2020–12.08.2021)	0.332	0.323	3.448	-0.885	9.158	20.457	20.459	0.975	0.005	2.083

To examine the reactions of investors to the arrival of unexpected information in the Bitcoin market, we first identified the date of the arrival of unexpected information. Contrary to previous studies [37, 53], we did not use economic and political news as surprises; rather, we employed a strictly quantitative approach to identify the market surprises. Specifically, following [67], we identified the market events by estimating GARCH(1,1) model for the data and defined thresholds for standardized residuals as 2.5 quantiles (as in [56]) of unexpected unfavorable information and, respectively, unexpected favorable information. Given the increasing number of studies employing a GARCH framework to measure normal returns within an event study (e.g. [68, 69]), we decided to use this model without predictors to obtain the standardized residuals. These are estimates of innovations normalized by their time-varying square root of the conditional variance and are more suitable in capturing outliers than the ordinary residuals. Thus, the resulting thresholds for the four periods under study obtained from the 2.5 quantiles of the standardized residuals for the unfavorable and favorable news are as follows: -2.198 and 2.015 (whole period), -2.017 and 2.108 (first period), -2.262 and 1.995 (second period), and -1.943 and 1.848 (COVID period). Then, by using these thresholds, we formed a set of 5-day windows where the starting point is the unexpected information day. We did not reckon with longer post-announcement windows since the cryptocurrency markets are highly volatile and react to the news very quickly [52]. This approach resulted in 79 windows following the arrival of bad news and 80 windows following the arrival of good news (whole period), 22 windows of negative events and 23 windows of positive events (first period), 48 windows following the arrival of unfavorable surprises and 46 windows following the arrival of favorable surprises (second period), and 12 window of bad news and 11 windows of good news (COVID19 period).

To assess whether the arrival of unexpected information increases the return volatility and risk in the Bitcoin market, we computed the variance of the daily returns of non-event days and post-event 5-day windows for the four analyzed periods. Afterward, using the F-test, we assayed whether the variance of returns over non-event days is higher compared to the variance of post-information windows. Concretely, if the arrival of unexpected information increases market volatility and risk, we would expect the variance of returns during post-information windows to be statistically significantly higher than the variance of returns for non-event days. The rejection of the null hypothesis provides evidence that the arrival of unexpected information causes a surge in Bitcoin market uncertainty at first, but the prices then follow a corrective upward trend.

To analyze investors’ reactions to the arrival of unexpected surprises in the Bitcoin market, we proceeded with the following steps of the well-known event study methodology, which is the main means of demonstrating how a market reacts to a signal. We believe that this approach is suitable for our research given its already proven statistical power, broad applicability in finance, and its continuous growing use [34, 70, 71]. However, we are aware of the limitations related to the methodology of an event study, and especially to one that does not deal with news announcements (e.g. [72, 73]), which is why we used two statistical tests for the significance of abnormal returns and changed the event window to strengthen the robustness.

First, we built a set of daily abnormal returns (AR_*t*_) of each day included in the 5-day post-information window. Following [53] and because the applicability of asset pricing models for cryptocurrency is still puzzling [60], we calculated the AR in a simple manner, as the difference between actual daily return and the expected return of each period–computed as the mean of non-event days. More specifically:

$${\text{AR}}_{t}={\text{R}}_{t}-{\text{R}}_{non}$$ (1)

where AR_*t*_ is the abnormal return for Bitcoin on day *t* following the arrival of unexpected information; *t* = 1,2,….5; R_*t*_ is the return of Bitcoin on day t in the 5-day-window; and R_*non*_ is the mean return for Bitcoin over the non-event days. This way, we look for a set of post-information windows containing daily abnormal returns.

Second, following the formation of abnormal returns windows, we computed the mean of the abnormal returns across each day *t* included in the 5-day-post-information windows, as follows:

$${\text{mean}\;\text{of}\;\text{AR}=1\text{M}}_{t}{\text{=1MAR}}_{t}$$ (2)

where *t* = 1, …M, M being the number of post-information windows formed after the arrival of unexpected events in each analyzed period.

Third, the mean abnormal returns are cumulated over post-information 5-day windows. Formally, the cumulative mean abnormal returns (CAR*t*) are generated as follows:

$${\text{CAR}}_{t}={\text{CAR}}_{\left(t-1\right)}+{\text{AR}}_{t}$$ (3)

Concisely, the OH prediction is verified if the CARs illustrate statistically significant downward (upward) trends following the arrival of unexpected favorable (unfavorable) information. Alternatively, the prediction of UIH is confirmed if the CARs show statistically significant positive or non-negative patterns during post-event windows.

Finally, because in the empirical literature unusual volume is used to evaluate the weak form efficiency of the traditional financial markets, we have incorporated in this research the analysis of the abnormal volume of this novel asset class, Bitcoin, to further improve the robustness of our results. In this regard, the abnormal trading volume methodology is an extension of our abnormal return methodology. Briefly, we computed the abnormal volume as the difference between the Bitcoin trading volume on day *t* of the event window and the average trading volume of Bitcoin over the non-event days. Similar to the return methodology, we calculated the cumulative abnormal volume (CAV) for the post-event windows.

To obtain robust results, two statistical tests have been applied to assess if the CARs and CAVs are statistically different from zero. The traditional parametric t-test has been conducted to test for the significance of CARs and CAVs over the event period. In addition, given the relatively small sample size and the already acknowledged nonnormality of the data, we also computed a nonparametric test statistic. Thus, the Wilcoxon sign rank test has been conducted to test the median significance of abnormal returns and trading volume.

## Results and discussion

Table 2 displays the means of daily returns for non-surprise days, post-arrival of unexpected information, post-favorable information days, and post-unfavorable information days. Briefly, as can be seen, the highest mean returns are registered within the first analyzed period. In addition, the results suggest that the average daily returns during the 5-day windows following favorable market surprises are greater in absolute terms than the ones registered within the post-unfavorable market surprises in all the cases.

**Table 2:** Mean Bitcoin daily returns for non-event and post-event days.

Period	Non-event days	All post-event days	Post-favorable event days	Post-unfavorable event days
Whole period (1.01.2011–12.08.2021)	0.2903	0.3751	2.9068	-2.1886
First period (1.01.2011–31.12.2013)	0.7582	0.5228	4.4376	-3.5343
Second period (1.01.2014–10.03.2020)	0.1209	0.0459	1.9628	-1.7911
COVID19 period (11.03.2020–12.08.2021)	0.2958	0.4591	2.4552	-1.3707

Post-event periods contain the days after both favorable and unfavorable events.

Table 3 contains the variance of returns for days on which there was no market surprise, all post-event days, post-favorable event days, and post-unfavorable event days. As can be seen, during all periods, the Bitcoin market volatility of returns appears to be higher in the post-event samples as opposed to non-event days, whereas the volatility of returns for the days following an unfavorable event is greater than the one for the days following a favorable event, except for the whole period under study. In addition, it is easy to remark that most of the F-values are significant at the 1% level–except for three cases–thus, verifying that the volatility of the Bitcoin market rises during the post-arrival of unexpected information. These results represent some insights that question the EMH, since the market uncertainty surges following the arrival of unexpected news.

**Table 3:** Variance of returns and F-test for Bitcoin.

Period	Sample	Variance	F-Value
Whole period (1.01.2011–12.08.2021)	Non-event days	9.3739	(a) 5.7056***
	All post-event days	53.4851	(b) 6.1546***
	Favorable	57.6935	(c) 3.8683***
	Unfavorable	36.2618	(d) 1.5910***
First period (1.01.2011–31.12.2013)	Non-event days	20.8851	(a) 5.3566***
	All post-event days	111.8734	(b) 4.5359***
	Favorable	94.7330	(c) 4.6915***
	Unfavorable	97.9847	(d) 1.0343
Second period (1.01.2014–10.03.2020)	Non-event days	5.4632	(a) 4.6440***
	All post-event days	25.3714	(b) 3.2807***
	Favorable	17.9231	(c) 4.7022***
	Unfavorable	25.6893	(d) 1.4333**
COVID19 period (11.03.2020–12.08.2021)	Non-event days	7.6720	(a) 3.5105***
	All post-event days	26.9330	(b) 2.3931***
	Favorable	18.3607	(c) 3.6647***
	Unfavorable	28.1161	(d) 1.5313*

(a) The F-statistic—marked a—tests the null hypothesis that the variance of returns for non-event days is equal to the variance of returns for all post-event days; (b) The F-statistic—marked b—tests the null hypothesis that the variance of returns after unexpected favorable events is equal to the variance of non-event returns; (c) The F-statistic—marked c—tests the null hypothesis that the variance of returns after unexpected unfavorable events is equal to the variance of non-event returns; (d) The F-statistic—marked d—tests the null hypothesis that the variance of returns after unexpected favorable events is equal to the variance of returns after unexpected unfavorable events.

***, **, * indicates statistical significance at the 1%, 5% and, respectively, 10% levels.

Table 4 presents the cumulative abnormal returns for the post-arrival of unexpected favorable and unfavorable information for the whole period (1.01.2011–12.08.2021), along with the t-values and z-values for the statistical significance tests. As the figures in Table 4 indicate, for all the analyzed event windows, the null hypotheses, i.e., that the mean and, respectively, the median of CARs are equal to zero, are rejected in the case of both parametric and nonparametric tests.

**Table 4:** Post-event CARs for the whole period (1.01.2011–12.08.2021)–favorable and unfavorable events.

	Favorable news			Unfavorable news		
Days	CARs	t-test	z-test	CARs	t-test	z-test
1	8.559	4.8787*	7.7700*	-8.692	-5.1886*	-7.7216*
2	12.015	6.8486*	7.7700*	-12.608	-7.5262*	-7.7216*
3	11.857	6.7586*	7.4918*	-12.302	-7.3435*	-7.6847*
4	12.392	7.0640*	7.0457*	-12.414	-7.4103*	-7.4626*
5	13.082	7.4572*	6.6620*	-12.395	-7.3989*	-7.2475*
Various event windows						
(0,2)	10.899	6.1371*	8.0501*	-12.406	-4.8604*	-7.9135*
(0,6)	12.557	4.3337*	7.2977*	-12.484	-7.7993*	-7.4245*

CARs, cumulative abnormal return.

For the T-test and the Wilcoxon sign rank test, the t-values and, respectively, z-values are provided.

* denotes statistical significance at the 10% level or higher.

The analysis of the whole period, which overlaps almost totally with the entire existence of Bitcoin, shows that the CARs exhibit a relatively upward trend at the arrival of unexpected favorable news. This suggests that the investors in the Bitcoin market, due to the uncertainly generated by the arrival of unexpected information, set the prices below their intrinsic value at first. However, as the certainty gradually settles, the Bitcoin prices track a corrective upward trend to some extent, approaching their intrinsic value, as postulated by the UIH. In the same vein, in the case of unfavorable news, the CARs exhibit a relatively non-negative trend, the fact that is in accordance with the UIH predictions. In other words, these findings reveal that the analysis conducted over the entire period shows evident signs of inefficiency within the Bitcoin market. These results are similar to those of [42], who found that historical cryptocurrencies are inefficient but register periods of efficiency. Therefore, the analysis of Bitcoin returns on smaller subsamples is essential.

Table 5 shows the cumulative abnormal returns following the arrival of unexpected favorable and unfavorable information and the t-values and z-values for the statistical significance tests for the first sub-period under investigation that corresponds to the emergence of Bitcoin. Just like in the above studied period, the results of the parametric and nonparametric tests are consistent for all the analyzed windows.

**Table 5:** Post-event CARs for the first sub-period (1.01.2011–31.12.2013)–favorable and unfavorable events.

	Favorable news			Unfavorable news		
Days	CARs	t-test	z-test	CARs	t-test	z-test
1	11.803	4.7437*	4.1973*	-12.473	-3.4377*	-4.1069*
2	16.201	6.5112*	4.1973*	-20.694	-5.7035*	-4.1069*
3	14.815	5.9541*	4.1668*	-19.074	-5.2569*	-4.0917*
4	16.757	6.7346*	4.1973*	-20.173	-5.5598*	-4.0744*
5	18.434	7.4087*	3.7410*	-21.462	-5.9152*	-4.0095*
Various event windows						
(0,2)	13.616	6.6216*	4.4573*	-18.247	-4.2230*	-4.2857*
(0,6)	18.376	3.5381*	4.0145*	-19.622	-5.6766*	-4.0145*

CARs, cumulative abnormal return.

For the T-test and the Wilcoxon sign rank test, the t-values and, respectively, z-values are provided.

* denotes statistical significance at the 10% level or higher.

In the first analyzed subsample, the behavior of Bitcoin prices seems to exhibit a relative increase in responses to the arrival of unexpected favorable information, an assumption which is consistent with the UIH predictions. However, in the case of unfavorable events, the fluctuant corrective pattern tends to show the presence of an overreacting behavior. We might mention here that there is a slight difference between these two hypotheses: while the OH provides evidence of investors irrationality due to bad news, the UIH assumes that investors respond rational to an increased volatility due to the arrival of good news. Therefore, as expected, within the early stages of Bitcoin, this market behaves in an inefficient manner. In this regard, a number of studies (e.g., [38, 41]) suggested the same lack of efficiency of Bitcoin during this interval.

Table 6 displays the cumulative abnormal returns following the arrival of unexpected favorable and unfavorable information and the corresponding t-values and z-values for the statistical significance tests for the second sub-period under study. The results of the tests are consistent for all the studied event windows, except for the last two days of the 5-day window. However, it became known that when parametric and nonparametric tests are applied, they frequently lead to different inferences [74].

**Table 6:** Post-event CARs for the second sub-period (1.01.2014–10.03.2020)–favorable and unfavorable events.

	Favorable news			Unfavorable news		
Days	CARs	t-test	z-test	CARs	t-test	z-test
1	5.977	4.4425*	5.9052*	-7.439	-5.1198*	-6.0308*
2	8.609	6.3988*	5.9052*	-10.921	-7.5162*	-6.0308*
3	8.721	6.4815*	3.3268*	-10.837	-7.4579*	-6.0152*
4	9.168	6.8141*	1.1199	-10.504	-7.2290*	-5.7129*
5	9.209	6.8446*	0.3441	-9.559	-6.5789*	-5.6308*
Various event windows						
(0,2)	7.962	6.0828*	5.7346*	-9.820	-4.7490*	-6.0927*
(0,6)	8.769	6.8251*	5.4617*	-9.581	-7.1451*	-4.9110*

CARs, cumulative abnormal return.

For the T-test and the Wilcoxon sign rank test, the t-values and, respectively, z-values are provided.

* denotes statistical significance at the 10% level or higher

From this point, the situation is starting to behave a bit differently if we look at the results of the second sub-period as compared to the previously analyzed data sets. Seemingly, the relative smooth trend along the last four days after the arrival of a positive surprise is cogent, which might imply that, within this interval, the result tends to sketch a moderate consistency with the prediction of the EMH. Regarding the negative surprises, it seems that the slight upward price reversal and adjustment in the last three days is consistent with the prediction of the overreaction hypothesis. In other words, it seems that the findings within the second period tend to emphasize a modest trend towards the efficiency of the Bitcoin market in the case of favorable news. Surprisingly, in the case of unfavorable news arrival, the graph indicates a persistent overreaction pattern of investors, yet featured by a more tranquil trend than in the previously studied periods. In line with [38, 43], our study holds that there are signs of enhanced efficiency of Bitcoin over time.

The cumulative abnormal returns following the arrival of unexpected favorable and unfavorable information and the t-values and z-values for the statistical significance tests for the COVID19 sub-period are shown in Table 7.

**Table 7:** Post-event CARs for the COVID19 sub-period (11.03.2020–12.08.2021)–favorable and unfavorable events.

	Favorable news			Unfavorable news		
Days	CARs	t-test	z-test	CARs	t-test	z-test
1	8.199	7.9154*	2.8306*	-9.490	-16.0627	-3.0159*
2	8.877	8.5703*	2.8306*	-8.777	-14.8558	-3.0159*
3	8.992	8.6804*	2.6219*	-8.600	-14.5567	-2.7957*
4	10.078	9.7294*	2.5638*	-7.891	-13.3563	-2.9512*
5	10.797	10.4230*	2.1017*	-8.332	-14.1027	-2.4216*
Various event windows						
(0,2)	8.587	22.5023*	3.4590*	-9.058	-21.4109*	-2.9580*
(0,6)	10.950	4.3035*	3.0127*	-7.972	-10.4482*	-2.5612*

CARs, cumulative abnormal return.

For the T-test and the Wilcoxon sign rank test, the t-values and, respectively, z-values are provided.

* denotes statistical significance at the 10% level or higher

As can be seen, the results exhibit a relatively stable pattern in Bitcoin returns starting with the day after the event, which implies the fact that investors’ behavior is consistent with the efficient market hypothesis, irrespective of the sign of the event. Therefore, in keeping with [74–77], we posit that during the pandemic, the Bitcoin market became more efficient. In addition, the abovementioned authors revealed that Bitcoin has similar efficiency with spot gold and is more efficient than S&P 500, MSCI World Index, and US Dollar Index during the pandemic, highlighting that this cryptocurrency is more resilient during an extreme event.

**Fig 1:**
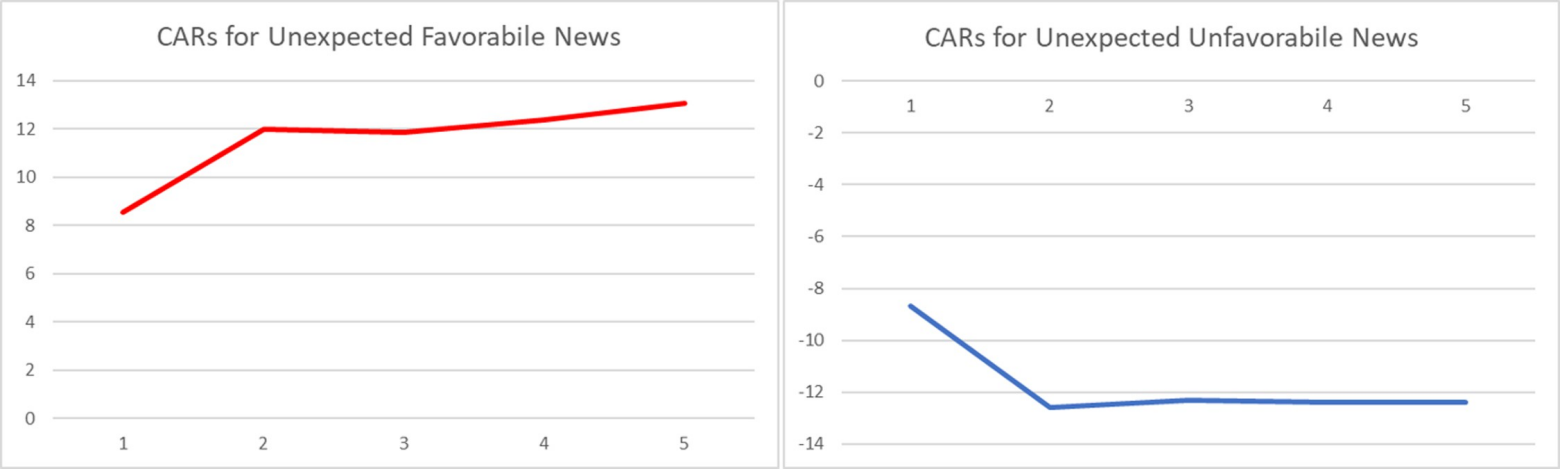
Graphs of daily CARs during a 5-day window following arrival of unexpected event (whole period– 1.01.2011 to 12.08.2021).

**Fig 2:**
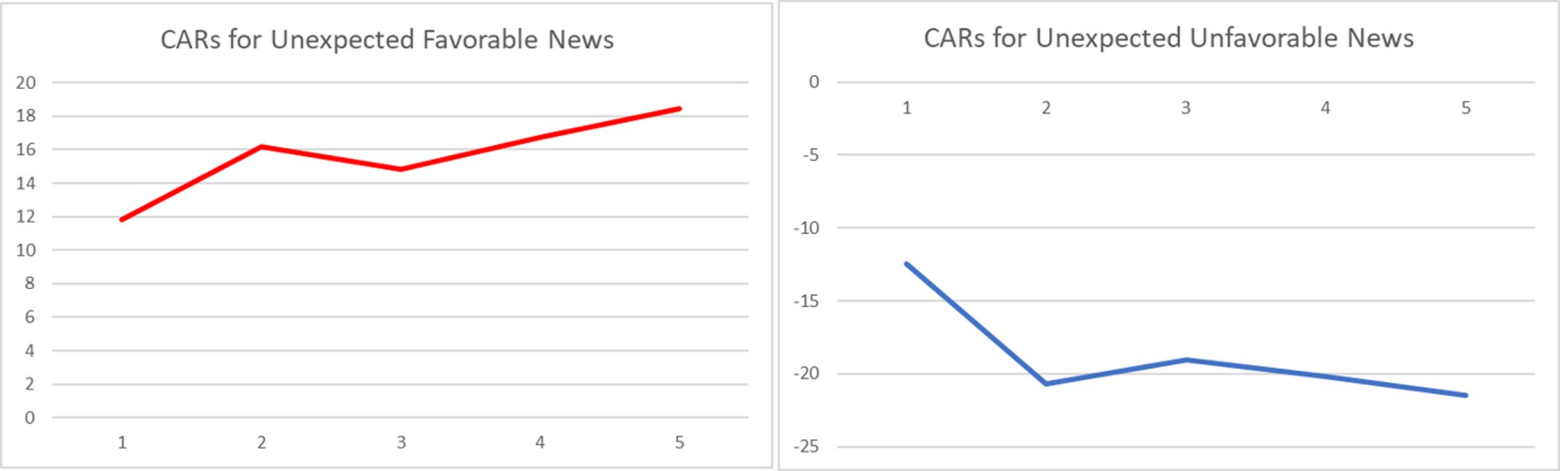
Graphs of daily CARs during a 5-day window following arrival of unexpected event (first period– 1.01.2011 to 31.12.2013).

**Fig 3:**
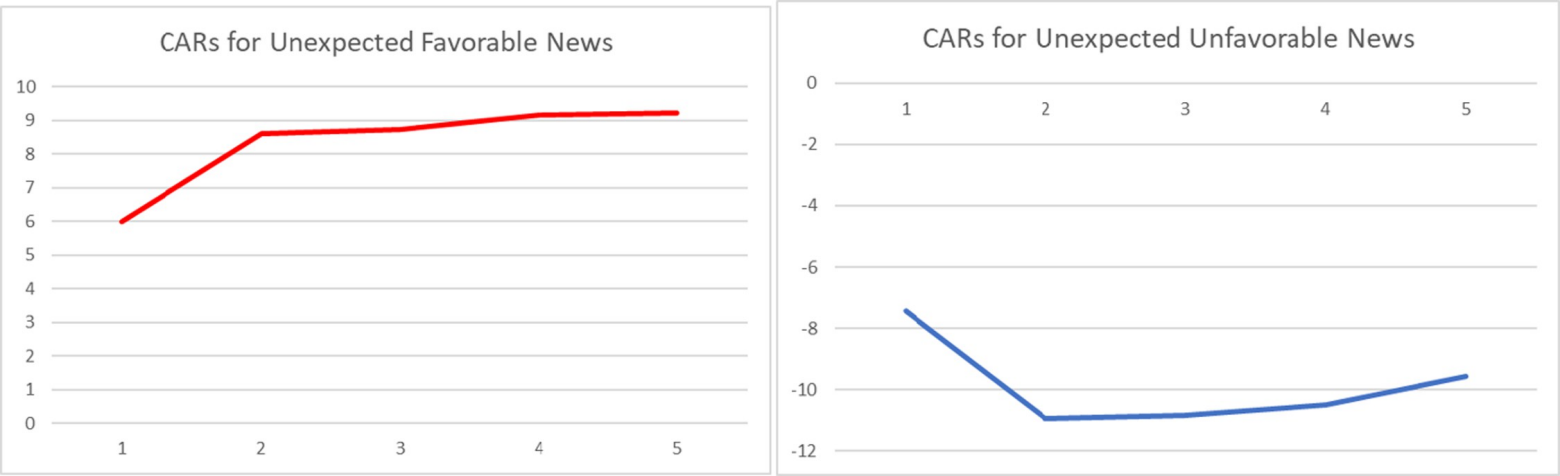
Graphs of daily CARs during a 5-day window following arrival of unexpected event (second period– 1.01.2014 to 10.03.2020).

**Fig 4:**
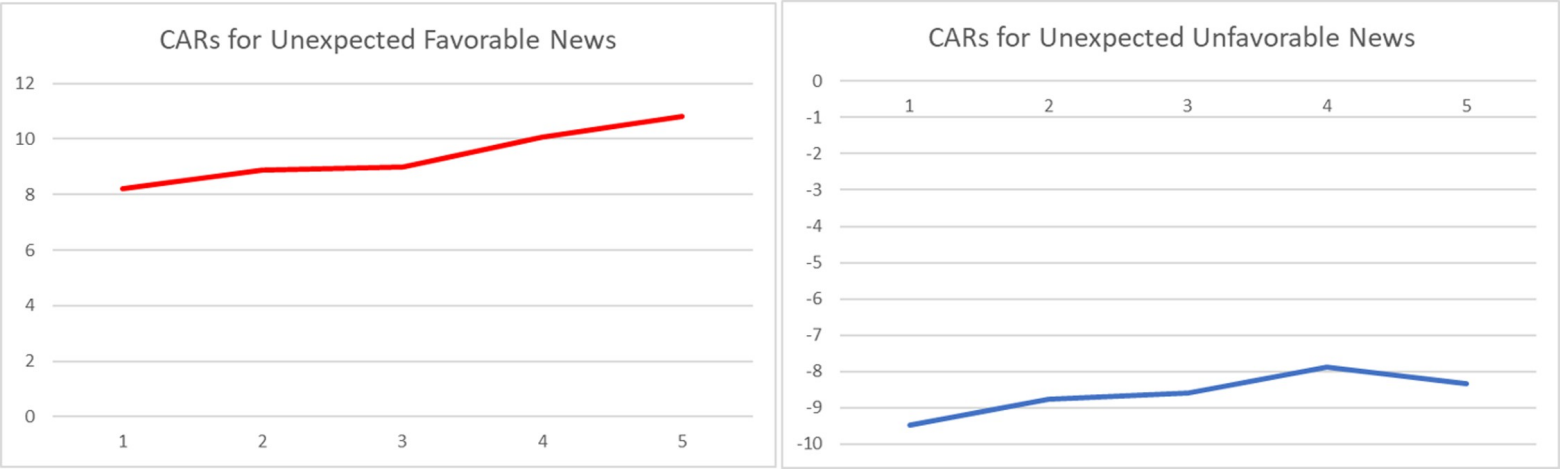
Graphs of daily CARs during a 5-day window following arrival of unexpected event (COVID19 period– 11.03.2020 to 12.08.2021).

The graphical representations of the CARs regarding the reactions of Bitcoin investors to the arrival of unexpected information are provided in Figs 1–4. The graphs visually verify the above discussion; more especially, within the four periods that we considered, high abnormal returns are noticed on the event day, suggesting that there exists a market reaction to surprises.

Comparing the magnitudes of CARs for negative and positive events, we can state that the market reacts stronger to negative surprises than to positive ones, especially within the first and the second sub-periods. The asymmetric behavior of this emerging asset is in line with the findings of [49, 51, 53]. However, the empirical evidence presented within Tables 4–7 suggests that the fluctuation of returns registered after the arrival of an unexpected event shows a decrease in magnitude over time.

The rationale of this improving efficiency and, respectively, lower volatility observed in the second subsample could be the increasing familiarity of investors with this particular market. Likewise, the variation in the efficiency of the market for Bitcoin dependent on the type of event was of interest in [37] who suggested that positive news appeared to increase the efficiency whilst negative news reduced the efficiency.

In addition, as can be seen in Tables 4–7, the average cumulative abnormal returns over two different lengths of windows were employed to strengthen the robustness of our results. The results across alternative event windows support our findings and, moreover, all reach statistical significance. The results indicate that if investors keep this particular asset after the occurrence of a favorable event, one can gain a substantial CAR and vice-versa for the unfavorable events. Thus, it seems that the short-term impacts of unexpected positive and negative news are noteworthy for the investors in the Bitcoin market. However, even though the results seem to be quite robust, the abnormal returns methodology merely reflects the beliefs of investors in a consensus price, thus, further empirical examination that incorporates volume trading is needed to better understand the reaction of investors to surprises.

Table 8 presents the cumulative abnormal trading volume following the arrival of unexpected favorable and unfavorable information and the t-values and z-values for the statistical significance tests for all the analyzed periods and event windows. Here, the results of the parametric and nonparametric tests are equivocal and more puzzling than in the abnormal return analysis.

**Table 8:** Post-event CAVs after unexpected favorable and unfavorable events, for the four analyzed periods.

	Favorable news			Unfavorable news		
	**Whole period** (1.01.2011–12.08.2021)					
**Days**	**CAVs**	**t-test**	**z-test**	**CAVs**	**t-test**	**z-test**
1	0.451	8.8286*	1.6307*	0.867	1.5221	3.1326*
2	0.552	10.7995*	1.2518	1.412	2.4785*	2.9567*
3	0.541	10.5851*	0.7866	1.794	3.1492*	2.8345*
4	0.567	11.0993*	0.6619	2.062	3.6207*	2.6341*
5	0.581	11.3700*	0.6475	2.317	4.0684*	2.5022*
Various Event Windows						
(0,2)	0.250	2.3262*	0.7428	0.797	4.1816*	2.5107*
(0,6)	1.242	2.7440*	1.3307	1.279	3.9958*	2.2284*
	**First sub-period** (1.01.2011–31.12.2013)					
**Days**	**CAVs**	**t-test**	**z-test**	**CAVs**	**t-test**	**z-test**
1	1.113	1.0245	2.0986*	-0.670	-0.4553	-0.6980
2	1.779	1.6382*	1.9161*	-1.425	-0.9687	-0.7305
3	2.300	2.1179*	1.8249*	-2.378	-1.6170*	-0.8279
4	3.019	2.7800*	1.7945*	-3.361	-2.2858*	-0.9577
5	3.912	3.6017*	1.7641*	-4.346	-2.9551*	-1.0227
Various Event Windows						
(0,2)	1.664	3.0195*	2.1715*	-2.271	-1.8069*	-1.2857
(0,6)	3.900	1.8435*	2.2419*	-4.399	-1.6315*	-1.1296
	**Second sub-period** (1.01.2014–10.03.2020)					
**Days**	**CAVs**	**t-test**	**z-test**	**CAVs**	**t-test**	**z-test**
1	0.922	1.0053	3.6873*	1.209	1.1671	4.2565*
2	1.589	1.7319*	3.2394*	2.032	1.9616*	4.0308*
3	2.166	2.3615*	2.7477*	2.749	2.6532*	3.8257*
4	2.768	3.0181*	2.5729*	3.322	3.2062*	3.6103*
5	3.228	3.5188*	2.2561*	3.824	3.6907*	3.4975*
Various Event Windows						
(0,2)	1.594	2.5519*	3.3771*	1.950	2.5990*	4.0436*
(0,6)	2.852	2.2630*	3.0343*	3.142	2.4482*	3.4251*
	**COVID19 sub-period** (11.03.2020–12.08.2021)					
**Days**	**CAVs**	**t-test**	**z-test**	**CAVs**	**t-test**	**z-test**
1	1.238	1.4450	1.9254*	1.618	1.2128	2.0309*
2	2.010	2.3452*	1.9017*	2.685	2.0130*	2.0185*
3	2.578	3.0089*	1.7519*	3.493	2.6182*	1.9867*
4	2.910	3.3958*	1.7361*	4.156	3.1151*	1.9760*
5	3.473	4.0533*	1.6845*	5.089	3.8148*	1.7645*
Various Event Windows						
(0,2)	1.813	2.9797*	1.9577*	2.470	2.8188*	2.1580*
(0,6)	2.790	2.6179*	1.7127*	3.857	2.3391*	1.6612*

CAVs, cumulative abnormal log trading volume.

For the T-test and the Wilcoxon sign rank test, the t-values and, respectively, z-values are provided.

* denotes statistical significance at the 10% level or higher

Briefly, the figures in Table 8 verifies the hypothesis that a surprise, whether negative or positive, is followed by an increase in trading activity, except for the first studied period in which the volume decreases in response to unfavorable news. Abnormal trading volume increases sharply on the day of the event, but afterwards it declines. There are only two exceptions to this pattern. On one hand, in the COVID19 period, after a slight drop in transaction volume, from day 4 this decrease is reverted and remains persistent even after 7 days. This implies that within this period, an unexpected positive or negative news has a lasting impact on Bitcoin trading activity. Also, as expected, during this last investigated period, the initial effect on trading volume is the largest of all analyzed periods. This could suggest that the price changes reflect more quickly the existing information to market participants, thus, emphasizing an improvement in the weak form efficiency of Bitcoin. On the other hand, the other exception was found in the first period. Here, in reaction to a favorable surprise, the volume reverts after a sharp decline and begins to increase from day 3. However, in this case that encompass the early stages of Bitcoin, we cannot speak about enhanced efficiency. A possible explanation could be related to the wide presence of uninformed investors in this newly emerging market. Furthermore, when we compare the magnitude of abnormal trading volume, it seems that investors react more strongly to negative surprises than to positive ones. Our results are in line with those of [61] who found significant abnormal volume on the cryptocurrency market. However, contrary to these results, we found larger reactions to the bad news than to the positive ones.

## Concluding remarks

Cryptocurrencies have become a buzzword on a global scale and are presently a fashionable topic in empirical financial research. In this regard, the study of cryptocurrency price behavior is of current relevance since the empirical evidence shows us that Bitcoin is becoming a far-reaching and increasingly worthy of consideration method of payment all around the world, and not just in anyway. Only in the last year, this crypto mania was embraced by giant companies such as Tesla, PayPal, AXA, and Bank of New York Mellon, and it seems that this fairy tale is to be continued.

Therefore, in this paper, we examined investors’ reactions to unexpected information with respect to the most popular cryptocurrency, Bitcoin, in an event study framework. The analysis of the entire interval suggests that investors’ reaction to favorable and unfavorable events is in line with the UIH. The results of the first sub-period, which encompasses the early stages of Bitcoin development, appear to be consistent with the UIH regarding the arrival of positive news and the OH regarding the arrival of negative news. The results of the second analyzed subsample show, with a slight vigor, some signs of improved efficiency in the case of positive events and a persistent overreaction of investors in the case of negative events. In other words, within these three abovementioned periods, significant price reversals exist in response to the arrival of unexpected events. Finally, the analysis of the pandemic period reveals an improved efficiency of the Bitcoin market. In accordance with these demonstrated behavioral hypotheses, our findings suggest that in the case of positive events, investors seem to act more rationally, while in the case of negative events, investors tend to behave a bit more irrational. Even more, the analysis reveals an asymmetric behavior of Bitcoin prices. The improved market efficiency, along with the asymmetric behavior of this cryptocurrency, is also supported by the abnormal trading volume analysis.

In summary, in line with the existing literature, our results generally suggest that investors’ behavior pictures an enhanced efficiency over time as the Bitcoin market matures and develops. Moreover, it seems that the pandemic period was favorable for Bitcoin efficiency.

Our findings are relevant for investors, especially for understanding the dynamics of cryptocurrencies over time and making informed investment decisions. Related to recent times, it seems that the increasing Bitcoin market efficiency proves the fact that this cryptocurrency cannot be utilized to generate remarkable abnormal returns. Moreover, this propriety acquired especially during the pandemic suggests that it may serve as a viable option for portfolio diversification and, maybe, as a safe haven. But even though the COVID period was favorable for the Bitcoin market, this exuberance should be moderated because the additional analysis that involves longer time periods is necessary.

Our analysis exhibits three main caveats: (1) the event study methodology based on a simple model to compute the expected return and trading volume; (2) the short event windows employed–a choice made due to the fact that this global market reacts rapidly to information and is highly volatile–which allowed us to draw conclusions mainly in the short term; (3) the daily frequency of the Bitcoin data used. The results presented in this paper pave the way to further investigations into the efficiency of cryptocurrency markets; for example, what are the factors that influence the market (in)efficiency during normal and turbulent times? Does this in vogue trend of acceptance as a means of payment by big companies improve the efficiency of this market?

## Supporting information

S1 Appendix Descriptive statistics for Bitcoin prices. (DOCX)

S2 Appendix Descriptive statistics for Bitcoin trading volume. (DOCX)
