# Supplementary material for: An analysis of investors’ behavior in Bitcoin market
Source: PLoS One. 2022 Mar 10;17(3):e0264522. doi: 10.1371/journal.pone.0264522 (PMC8912198; doi:10.1371/journal.pone.0264522)
Supplement: S1 Appendix — (DOCX) [file pone.0264522.s001.docx]

S1 Descriptive statistics for Bitcoin prices

| **Statistic** | **Whole period**  (01.01.2011-12.08.2021) | **First period (**01.01.2011-31.12.2013) | **Second period** (1.01.2014-10.03.2020) | **COVID19 period** (11.03.2020-12.08.2021) |
| --- | --- | --- | --- | --- |
| Mean | 5523.220 | 67.30747 | 3550.218 | 25590.87 |
| Median | 623.0268 | 10.91829 | 1003.932 | 18284.46 |
| Max. | 63414.96 | 1119.794 | 19389.51 | 63414.96 |
| Min. | 0.293518 | 0.293518 | 198.9046 | 5053.947 |
| Std.dev. | 10750.55 | 163.5642 | 3898.369 | 17703.92 |
| Skewness | 3.200161 | 4.136710 | 1.017324 | 0.581437 |
| Kurtosis | 13.73007 | 20.78929 | 3.129244 | 1.895378 |
| Obs. | 3876 | 1095 | 2261 | 520 |
