# Supplementary material for: An analysis of investors’ behavior in Bitcoin market
Source: PLoS One. 2022 Mar 10;17(3):e0264522. doi: 10.1371/journal.pone.0264522 (PMC8912198; doi:10.1371/journal.pone.0264522)
Supplement: S2 Appendix — (DOCX) [file pone.0264522.s002.docx]

S2 Descriptive statistics for Bitcoin trading volume

| **Statistic** | **Whole period**  (01.01.2011-12.08.2021) | **First period (**01.01.2011-31.12.2013) | **Second period** (1.01.2014-10.03.2020) | **COVID19 period** (11.03.2020-12.08.2021) |
| --- | --- | --- | --- | --- |
| Mean | 313176878.044 | 6035792.643 | 258281327.657 | 1198635201.002 |
| Median | 37677125.510 | 389696.184 | 72159168.097 | 768028024.513 |
| Max. | 9988780995.633 | 227901342.774 | 5457602193.998 | 9988780995.633 |
| Min. | 0.000 | 0.000 | 2750898.110 | 91321114.126 |
| Std.dev. | 664006650.671 | 20648284.416 | 444365287.881 | 1197274514.782 |
| Skewness | 4.486807 | 6.447429 | 3.971645 | 2.326983 |
| Kurtosis | 34.73369 | 54.12460 | 27.00736 | 12.00689 |
| Obs. | 3876 | 1095 | 2261 | 520 |
